# Appropriate number of observations for determining hand hygiene compliance among healthcare workers

**DOI:** 10.1186/s13756-021-01035-1

**Published:** 2021-12-02

**Authors:** Se Yoon Park, Suyeon Park, Beom Seuk Hwang, Eunjung Lee, Tae Hyong Kim, Sungho Won

**Affiliations:** 1 Division of Infectious Diseases, Department of Internal Medicine, Soonchunhyang University, Seoul Hospital, Soonchunhyang University College of Medicine, 59, Daesagwan-ro, Yongsan-gu, Seoul, 04401 Republic of Korea; 2 Department of Biostatistics, Soonchunhyang University Seoul Hospital, Seoul, Republic of Korea; 3 Department of Applied Statistics, Chung-Ang University, Seoul, Republic of Korea; 4 Department of Public Health Science, Seoul National University, Seoul, 08826 Republic of Korea

**Keywords:** Hand hygiene, Monitor, Healthcare worker, Compliance, Observation

## Abstract

We sought to determine the minimum number of observations needed to determine hand hygiene (HH) compliance among healthcare workers. The study was conducted at a referral hospital in South Korea. We retrospectively analyzed the result of HH monitoring from January to December 2018. HH compliance was calculated by dividing the number of observed HH actions by the total number of opportunities. Optimal HH compliance rates were calculated based on adherence to the six-step technique recommended by the World Health Organization. The minimum number of required observations (n) was calculated by the following equation using overall mean value (ρ), absolute precision (*d*), and confidence interval (CI) (1 − α) [the equation: ${\text{n}} \ge Z_{\alpha /2}^{2} \times \rho \times \left( {1 - \rho } \right)/d^{2}$]. We considered *d*s of 5%, 10%, 20%, and 30%, with CIs of 99%, 95%, and 90%. During the study period, 8791 HH opportunities among 1168 healthcare workers were monitored. Mean HH compliance and optimal HH compliance rates were 80.3% and 59.7%, respectively. The minimum number of observations required to determine HH compliance rates ranged from 2 ($d$: 30%, CI: 90%) to 624 ($d$: 5%, CI: 99%), and that for optimal HH compliance ranged from 5 ($d$: 30%, CI: 90%) to 642 ($d$: 5%, CI: 99%). Therefore, we found that our hospital required at least five observations to determine optimal HH compliance.

**Supplementary Information:**

The online version contains supplementary material available at 10.1186/s13756-021-01035-1.

## Background

Hand hygiene (HH) is known to be one of the most basic and effective strategies for preventing healthcare-associated infections [1]. HH can prevent the spread of pathogens between healthcare workers (HCWs) and patients, between HCWs themselves, and between the environment and HCWs. Medical institutions can determine the levels and quality of HH among their HCWs through HH monitoring. The HH compliance rate serves as an indicator of healthcare-associated infection rates and can be used to develop strategies for HH promotion and to determine the most appropriate intervention activities, such as education and training [2].

To perform a meaningful before-and-after comparison regarding the improvement effects of HH interventions, the World Health Organization (WHO) manual for HH observation recommends observing a minimum of 200 opportunities for HH in each department or ward during each measurement period [3]. This ensures that the number of observations is sufficient to draw valid conclusions within groups. However, it should be noted that 200 is not an exact or required number for actual observations. Yin et al. estimated that between 79 and 723 opportunities must be measured during each period on the basis of an improvement goal and target compliance rate [4]. It is also difficult to know the appropriate number of HH observations for each individual. In the present study, we aimed to determine, based on actual results of HH monitoring, the number of observations needed to estimate HH compliance.

## Methods and materials

### Study setting and design

This study was conducted in Soonchunhyang University Seoul Hospital, a 734-bed acute-care referral hospital in South Korea. It was approved by the Institutional Review Board (approval number: 2019-01-008). Since 2010, we have maintained an HH monitoring team at the hospital, comprising 24 members across various departments; the infection control team comprises five members. Every quarter, approximately 2500 HH opportunities are monitored by the infection control and HH monitoring team members. We trained HH monitor personnel on monitoring methods, precautions on observation, input of results, and practice through monthly meetings. In the case of the existing monitoring team, we maintained the quality of monitoring by conductiong video training and testing the HH monitors at the first meeting of the year. We follow standard HH monitoring methods by directly observing HH per WHO guidelines [1]. The HH monitor was conducted during the observer’s working hours and there were no restrictions during the week days, weekend, day and night. In order to prevent the Hawthorne effect, observations for one HCW were limited to less than four, and the observation time per department was limited to less than 20 min [4]. Observers in each department did not monitor members of their own department. From January to December 2018, we collected data regarding the HH compliance rates of doctors, nurses, and other HCWs (medical technical assistants, dieticians, physiotherapists, and radiological technologists).

### Statistical analysis

The HH compliance rate was calculated by dividing the number of observed HH actions by the total number of opportunities. Opportunities were defined based on the WHO’s “5 moments for HH” (before touching a patient, after touching a patient, before clean/aseptic procedures, after body fluid exposure/risk, and after touching patients’ surroundings). Meanwhile, rates of compliance with optimal HH techniques were calculated based on adherence to the six-step technique recommended by the WHO on each opportunity (rub hands palm to palm, right palm over left dorsum with interlaced fingers, and vice versa; palm to palm with fingers interlaced; backs of fingers to opposing palms with fingers interlocked; rotational rubbing of left thumb clasped in the right palm, and vice versa; and rotational rubbing, backward and forwards, with clasped fingers of the right hand in left palm, and vice versa) [1, 5].

The HH compliance/optimal HH compliance values were calculated for each observed person and the data were expressed as mean, median, and interquartile range (IQR) measurements. We used the generalized estimating equation model for logistic regression using an unstructured working correlation matrix to compare HH compliance or optimal HH compliance rates in different job categories (doctors, nurses, and other HCWs) and year quarters.

To calculate the sample size for estimating the population’s HH compliance and optimal HH compliance, the following conditions were considered: (1) the variability in the target population; (2) the desired precision in the estimate; and (3) the desired confidence in the estimate. In this study, the following equation was applied:

$${\text{n}} \ge Z_{\alpha /2}^{2} \times \rho \times \left( {1 - \rho } \right)/d^{2} ,$$

where ρ represents the population proportion, *d* the absolute difference, and 1-α the confidence interval (CI) [6, 7]. This sample size can be interpreted as the minimum sample size required to get the sample proportion to fall within 100*d*% of the true proportion with 100(1 − α)% probability. We considered *d*s of 5%, 10%, 20%, and 30%, with CIs of 99%, 95%, and 90%, respectively. Among the various cases, we focused on 10% for *d* and 95% for CI. We calculated the number of n using the R package (‘binomSamSize’) and selected three methods to represent them in a Additional file 1: Table S1. The first method approximation is based on the central limit theorem [8] The other two are the Wilson score method [9] and the Agresti-Coull method [10], which can be used even when the data are asymmetric, the sample is small, and the observations are biased [11].

## Results

During the study period, 8791 HH opportunities among 1137 HCWs (574 nurses, 321 doctors, and 242 others) were monitored. Mean rates of compliance for HH and optimal HH were 80.3% and 59.7%, respectively (Table 1). Throughout the study period (one year), the median number of observations per HCW was five (IQR: 2–10, range: 1–74 observations).

**Table 1:** Mean hand hygiene and optimal hand hygiene compliance in terms of job category and year quarter

	Number of observations	Healthcare workers	Mean, median (IQR) HH compliance	*p *value^a^	Mean, median (IQR) optimal HH compliance	*p *value^†^
Total	8791	1137	80.3, 100 (66.7–100)		59.7, 75 (0–100)	
Job category				< 0.001		< 0.001
Nurse	4090	574	90.9, 100 (100–100)		78.6, 100 (62.5–100)	
Doctor	2843	321	62.2, 71.4 (33.3–100)		27.6, 0 (0–50)	
Other	1858	242	80.8, 100 (66.7–100)		60.2, 75 (0–100)	
Quarter				0.011		< 0.001
First	2586	615	80.0, 100 (66.7–100)		59.6, 72.7 (0–100)	
Second	1805	598	78.9, 100 (60–100)		59.8, 80 (0–100)	
Third	2352	673	78.8, 100 (66.7–100)		59.1, 75 (0–100)	
Fourth	2048	621	83.7, 100 (80–100)		60.6, 80 (0–100)	

*IQR* interquartile range, *HH* hand hygiene

^a^*p* value determined through generalized estimating equation

The minimum number of observations required to determine HH compliance rates ranged from two ($d$: 30%, CI: 90%) to 624 ($d$: 5%, CI: 99%), and that for optimal HH compliance ranged from five ($d$: 30%, CI: 90%) to 642 ($d$: 5%, CI: 99%). At 10% absolute precision with 95% confidence, the minimum number of observations to determine HH and optimal HH compliance were 61 and 92, respectively.

**Fig. 1:**
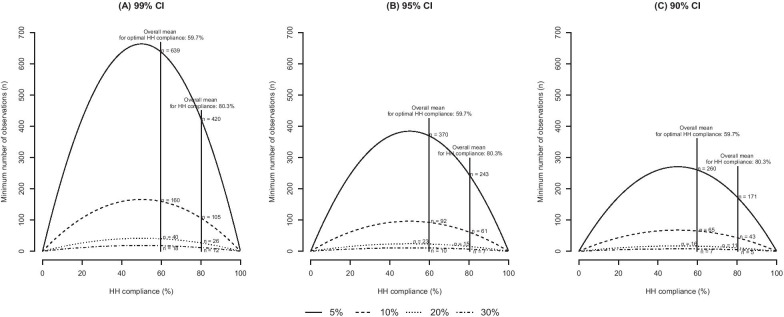
Minimum number of observations for determining hand hygiene compliance for absolute precisions of 5, 10, 20, and 30%, at confidence intervals of **a** 99%, **b** 95%, and **c** 90%

In terms of job category, sample means of (optimal) HH compliance for nurses and doctors were 90.9% (78.6%) and 62.2% (27.6%), respectively. If we used those values, using a *d* of 10% and applying 99%, 95%, and 90% CIs, respectively, the minimum number of observations required to determine HH compliance was 55, 32, and 22 for nurses, 156, 90, and 64 for doctors, and 103, 60, and 42 for other HCWs. Meanwhile, regarding optimal HH, the minimum number of observations was 112, 65, and 46 for nurses, 133, 77, and 54 for doctors, and 159, 92, and 65 for other HCWs, respectively (Fig. 1, Additional file 1: Table S1).

## Discussion

Through observing HH at a large medical institution over year, this research determined the minimum number of HH observations required to appropriately monitor HH compliance. Although, the required number of observations changed depending on the settings for *d* and CI. For a *d* of 10% and a CI of 95%, the minimum number of observations to estimate the overall mean of HH compliance and optimal HH compliance were 61 and 92, respectively. To our knowledge, this study is the first to provide data verifying the appropriate number of observations for determining HH compliance.

HH compliance rates reported in previous literature have been based on analysis of compliance rates in terms of job category without any lower limit on the number of observations per person [2, 5, 12]. Typically, the observation numbers in these studies were only two to four per medical personnel approximately [5, 12]. Similarly, in the present study, the number of observations among each medical personnel ranged from one to hundreds. However, our study shows that optimal HH compliance requires at least five observations per person, and up to 624 are required for elaborate calculations. In the case of medical staff for which there are few opportunities to observe HH, a higher number of observations may be necessary to ensure accurate evaluation; alternatively, a method other than direct observation could be used for monitoring [13]. Moreover, we suggest that each medical institution should determine the minimum number of observations to be applied using statistics, and the criteria may be individualized for each job category and compliance rate.

There are some limitations to this study. First, it was conducted in a single acute-care hospital using direct observation methods. The appropriate number of observations for determining HH compliance rate can differ depending on the characteristics of the setting (i.e., the institution) and the methodology applied (i.e., the observation method), among other factors. Second, the direct observation method cannot exclude the effect of increasing compliance due to the Hawthorne effect. In order to minimize the Hawthorne effect, the number of observations for one HCW was only up to four, and the observation time per department was limited to less than 20 min.

## Conclusions and outlook

On the basis of our findings, we recommend that at least five opportunities should be monitored to determine individual optimal HH compliance and give feedback. When it is difficult to observe HH directly, such as inside the outpatient clinic or the place where the procedure is performed, the sufficient number of observations is limited. In such situations, it is suggested to monitor using an indirect method.

If each institution sets the minimum number of observations according to the performance rate of HH based on this study, unnecessary observations can be minimized. Accordingly, it is expected that the distribution of human resources for HH monitors will be effective. In addition, the findings are expected to be useful for HH observers and future HH-related studies, as they provide criteria for estimating the number of observations in general and in relation to specific job categories.

## Supplementary Information

**Additional file 1**. **Table 1:** Number of observations to determine hand hygiene compliance.

## Data Availability

All data generated or analyzed during this study are included in this published article and its supplementary files.

## Abbreviations

HH: Hand hygiene
HCWs: Healthcare workers
WHO: World Health Organization
IQR: Interquartile range
CI: Confidence interval

## References

[1] WHO guidelines on hand hygiene in health care. [cited 25 Jun 2021]. https://apps.who.int/iris/bitstream/handle/10665/44102/9789241597906_eng.pdf;jsessionid=6C30E8483C433B8C2978EA44B2D873E9?sequence=1. World Health Organization. p. 2009.

[2] Shim JY, Park S, Kim GE, Jeong YS, Kim JH, Lee E (2019). Does physician leadership influence followers’ hand hygiene compliance*?*. Open Forum Infect Dis.

[3] Sax H, Allegranzi B, Chraïti MN, Boyce J, Larson E, Pittet D (2009). The World Health Organization hand hygiene observation method. Am J Infect Control.

[4] Yin J, Reisinger HS, Vander Weg M, Schweizer ML, Jesson A, Morgan DJ (2014). Establishing evidence-based criteria for directly observed hand hygiene compliance monitoring programs: a prospective, multicenter cohort study. Infect Control Hosp Epidemiol.

[5] Baek EH, Kim SE, Kim DH, Cho OH, Hong SI, Kim S (2020). The difference in hand hygiene compliance rate between unit-based observers and trained observers for World Health Organization checklist and optimal hand hygiene. Int J Infect Dis.

[6] Daniel WW (1999). Biostatistics: a foundation for analysis in the health sciences.

[7] Lemeshow S, Hosmer DW, Klar J, Lwanga SK. Adequacy of sample size in health studies; 1990. [cited 25 Jun 2021]. http://apps.who.int/iris/bitstream/10665/41607/1/0471925179_eng.pdf?ua=1. Chichester, UK: Wiley.

[8] Brown LD, Cai TT, DasGupta A (2001). Interval estimation for a binomial proportion. Stat Sci.

[9] Wilson EB (1927). Probable inference, the law of succession, and statistical inference. J Am Stat Assoc.

[10] Agresti A, Coull BA (1998). Approximate is better than “exact” for interval estimation of binomial proportions. Am Stat.

[11] Wallis S (2013). Binomial confidence intervals and contingency tests: mathematical fundamentals and the evaluation of alternative methods. J Quant Linguist.

[12] Lee SS, Park SJ, Chung MJ, Lee JH, Kang HJ, Lee JA (2014). Improved hand hygiene compliance is associated with the change of perception toward hand hygiene among medical personnel. Infect Chemother.

[13] Kato H, Takeda R, Ideno Y, Suzuki T, Sano K, Nakamura K (2021). Physicians’ compliance for hand hygiene in medical outpatient clinics: automated hand-hygiene monitoring with touch sensor and wireless internet. Am J Infect Control.

