# Supplementary material for: Appropriate number of observations for determining hand hygiene compliance among healthcare workers
Source: Antimicrob Resist Infect Control. 2021 Dec 2;10:167. doi: 10.1186/s13756-021-01035-1 (PMC8638267; doi:10.1186/s13756-021-01035-1)
Supplement: Supplementary file 1 — Additional file 1. Table 1: Number of observations to determine hand hygiene compliance. [file 13756_2021_1035_MOESM1_ESM.docx]

**Supplemental Table 1.** Number of observations to determine hand hygiene compliance.

|  | | |  |  | 99% CI | | | | 95% CI | | | | 90% CI | | | |
| --- | --- | --- | --- | --- | --- | --- | --- | --- | --- | --- | --- | --- | --- | --- | --- | --- |
|  |  |  | Mean |  | 0.05 | 0.1 | 0.2 | 0.3 | 0.05 | 0.1 | 0.2 | 0.3 | 0.05 | 0.1 | 0.2 | 0.3 |
| All | | HH | 80.3 | Simple asymptotic | 420 | 105 | 26 | 12 | 243 | 61 | 15 | 7 | 171 | 43 | 11 | 5 |
|  |  |  |  | Score method1 | 418 | 103 | 24 | 9 | 242 | 60 | 14 | 5 | 171 | 42 | 10 | 4 |
|  |  |  |  | Score method2 | 414 | 99 | 20 | 6 | 240 | 57 | 12 | 3 | 169 | 41 | 8 | 3 |
|  |  | Optimal HH | 59.7 | Simple asymptotic | 639 | 160 | 40 | 18 | 370 | 92 | 23 | 10 | 260 | 65 | 16 | 7 |
|  |  |  |  | Score method1 | 633 | 154 | 34 | 12 | 366 | 89 | 20 | 7 | 258 | 63 | 14 | 5 |
|  |  |  |  | Score method2 | 632 | 153 | 34 | 12 | 366 | 89 | 20 | 7 | 258 | 63 | 14 | 5 |
| Job category | Nurse | HH | 90.9 | Simple asymptotic | 220 | 55 | 14 | 6 | 127 | 32 | 8 | 4 | 90 | 22 | 6 | 2 |
|  |  |  |  | Score method1 | 226 | 60 | 16 | 6 | 131 | 35 | 9 | 4 | 92 | 25 | 7 | 3 |
|  |  |  |  | Score method2 | 213 | 49 | 8 | 1 | 124 | 28 | 5 | 1 | 87 | 20 | 3 | 1 |
|  |  | Optimal HH | 78.6 | Simple asymptotic | 446 | 112 | 28 | 12 | 258 | 65 | 16 | 7 | 182 | 46 | 11 | 5 |
|  |  |  |  | Score method1 | 443 | 109 | 25 | 9 | 257 | 63 | 15 | 5 | 181 | 45 | 10 | 4 |
|  |  |  |  | Score method2 | 440 | 105 | 22 | 6 | 255 | 61 | 13 | 4 | 180 | 43 | 9 | 3 |
|  | Doctor | HH | 62.2 | Simple asymptotic | 624 | 156 | 39 | 17 | 361 | 90 | 23 | 10 | 254 | 64 | 16 | 7 |
|  |  |  |  | Score method1 | 618 | 150 | 33 | 12 | 358 | 87 | 19 | 7 | 252 | 62 | 14 | 5 |
|  |  |  |  | Score method2 | 618 | 150 | 33 | 11 | 358 | 87 | 19 | 7 | 252 | 61 | 14 | 5 |
|  |  | Optimal HH | 27.6 | Simple asymptotic | 530 | 133 | 33 | 15 | 307 | 77 | 19 | 9 | 216 | 54 | 14 | 6 |
|  |  |  |  | Score method1 | 526 | 128 | 29 | 10 | 305 | 74 | 17 | 6 | 215 | 53 | 12 | 4 |
|  |  |  |  | Score method2 | 524 | 126 | 27 | 9 | 304 | 73 | 16 | 5 | 214 | 52 | 11 | 4 |
|  | Other | HH | 80.8 | Simple asymptotic | 412 | 103 | 26 | 11 | 238 | 60 | 15 | 7 | 168 | 42 | 10 | 5 |
|  |  |  |  | Score method1 | 410 | 101 | 23 | 8 | 237 | 59 | 14 | 5 | 167 | 41 | 10 | 4 |
|  |  |  |  | Score method2 | 406 | 97 | 20 | 5 | 235 | 56 | 12 | 3 | 166 | 40 | 8 | 2 |
|  |  | Optimal HH | 60.2 | Simple asymptotic | 636 | 159 | 40 | 18 | 368 | 92 | 23 | 10 | 259 | 65 | 16 | 7 |
|  |  |  |  | Score method1 | 630 | 153 | 34 | 12 | 365 | 89 | 20 | 7 | 257 | 63 | 14 | 5 |
|  |  |  |  | Score method2 | 630 | 153 | 34 | 12 | 365 | 89 | 20 | 7 | 257 | 63 | 14 | 5 |
| Quarter | First | HH | 79.9 | Simple asymptotic | 426 | 107 | 27 | 12 | 247 | 62 | 15 | 7 | 174 | 43 | 11 | 5 |
|  |  |  |  | Score method1 | 424 | 104 | 24 | 9 | 246 | 60 | 14 | 5 | 173 | 43 | 10 | 4 |
|  |  |  |  | Score method2 | 420 | 100 | 21 | 6 | 243 | 58 | 12 | 4 | 172 | 41 | 9 | 3 |
|  |  | Optimal HH | 59.6 | Simple asymptotic | 639 | 160 | 40 | 18 | 370 | 92 | 23 | 10 | 261 | 65 | 16 | 7 |
|  |  |  |  | Score method1 | 633 | 154 | 34 | 12 | 367 | 89 | 20 | 7 | 258 | 63 | 14 | 5 |
|  |  |  |  | Score method2 | 633 | 154 | 34 | 12 | 367 | 89 | 20 | 7 | 258 | 63 | 14 | 5 |
|  | Second | HH | 78.9 | Simple asymptotic | 442 | 110 | 28 | 12 | 256 | 64 | 16 | 7 | 180 | 45 | 11 | 5 |
|  |  |  |  | Score method1 | 439 | 108 | 24 | 9 | 254 | 62 | 14 | 5 | 179 | 44 | 10 | 4 |
|  |  |  |  | Score method2 | 436 | 104 | 21 | 6 | 252 | 61 | 13 | 4 | 178 | 43 | 9 | 3 |
|  |  | Optimal HH | 59.8 | Simple asymptotic | 638 | 160 | 40 | 18 | 369 | 92 | 23 | 10 | 260 | 65 | 16 | 7 |
|  |  |  |  | Score method1 | 632 | 154 | 34 | 12 | 366 | 89 | 20 | 7 | 258 | 63 | 14 | 5 |
|  |  |  |  | Score method2 | 632 | 153 | 34 | 12 | 366 | 89 | 20 | 7 | 258 | 63 | 14 | 5 |
|  | Third | HH | 78.8 | Simple asymptotic | 443 | 111 | 28 | 12 | 257 | 64 | 16 | 7 | 181 | 45 | 11 | 5 |
|  |  |  |  | Score method1 | 440 | 108 | 25 | 9 | 255 | 63 | 14 | 5 | 180 | 44 | 10 | 4 |
|  |  |  |  | Score method2 | 437 | 105 | 22 | 6 | 253 | 61 | 13 | 4 | 179 | 43 | 9 | 3 |
|  |  | Optimal HH | 59.1 | Simple asymptotic | 642 | 160 | 40 | 18 | 371 | 93 | 23 | 10 | 262 | 65 | 16 | 7 |
|  |  |  |  | Score method1 | 636 | 154 | 34 | 12 | 368 | 90 | 20 | 7 | 259 | 63 | 14 | 5 |
|  |  |  |  | Score method2 | 635 | 154 | 34 | 12 | 368 | 90 | 20 | 7 | 259 | 63 | 14 | 5 |
|  | Fourth | HH | 83.7 | Simple asymptotic | 362 | 91 | 23 | 10 | 210 | 52 | 13 | 6 | 148 | 37 | 9 | 4 |
|  |  |  |  | Score method1 | 361 | 90 | 21 | 8 | 209 | 52 | 12 | 5 | 148 | 37 | 9 | 4 |
|  |  |  |  | Score method2 | 356 | 84 | 16 | 4 | 206 | 49 | 10 | 2 | 145 | 35 | 7 | 2 |
|  |  | Optimal HH | 60.6 | Simple asymptotic | 634 | 158 | 40 | 18 | 367 | 92 | 23 | 10 | 258 | 65 | 16 | 7 |
|  |  |  |  | Score method1 | 628 | 153 | 34 | 12 | 364 | 89 | 20 | 7 | 256 | 63 | 14 | 5 |
|  |  |  |  | Score method2 | 628 | 152 | 33 | 11 | 364 | 88 | 20 | 7 | 256 | 62 | 14 | 5 |
| CI, confidence interval; HH, hand hygiene. | | | |  |  |  |  |  |  |  |  |  |  |  |  |  |
| Score method1 is developed by Wilson score method. | | | | |  |  |  |  |  |  |  |  |  |  |  |  |
| Score method2 is developed by Agresti–Coull method. | | | | |  |  |  |  |  |  |  |  |  |  |  |  |
